# Targeting Siglec–Sialylated MUC1 Immune Axis in Cancer

**DOI:** 10.3390/cancers16071334

**Published:** 2024-03-29

**Authors:** Ramya Ayyalasomayajula, Mare Cudic

**Affiliations:** Department of Chemistry and Biochemistry, Florida Atlantic University, 777 Glades Rd., Boca Raton, FL 33431, USA; rayyalasomay2017@fau.edu

**Keywords:** MUC1, Siglecs, sialoglycans, glycocode, immune evasion

## Abstract

**Simple Summary:**

In this review, we will focus on the interactions between tumor-associated MUC1 (cell-surface mucin) and Siglecs (sialic-acid-binding lectins). These interactions play a central role in the evasion of antitumor immune responses. Tumor cells utilize this mechanism to either evade immune cell detection or inhibit the antitumor immune response. Thus, interference with sialoglycan–Siglec interactions could represent a new immune checkpoint and a potential new target for cancer immunotherapy.

**Abstract:**

Siglecs play a key role in mediating cell–cell interactions via the recognition of different sialylated glycoconjugates, including tumor-associated MUC1, which can lead to the activation or inhibition of the immune response. The activation occurs through the signaling of Siglecs with the cytoplasmic immunoreceptor tyrosine-based activation motif (ITAM)-containing proteins, while the inhibition signal is a result of the interaction of intracellular immunoreceptor tyrosine-based inhibition motif (ITIM)-bearing receptors. The interaction of tumor-associated MUC1 sialylated glycans with Siglecs via ITIM motifs decreases antitumor immunity. Consequently, these interactions are expected to play a key role in tumor evasion. Efforts to modulate the response of immune cells by blocking the immune-suppressive effects of inhibitory Siglecs, driving immune-activating Siglecs, and/or altering the synthesis and expression of the sialic acid glycocalyx are new therapeutic strategies deserving further investigation. We will highlight the role of Siglec’s family receptors in immune evasion through interactions with glycan ligands in their natural context, presented on the protein such as MUC1, factors affecting their fine binding specificities, such as the role of multivalency either at the ligand or receptor side, their spatial organization, and finally the current and future therapeutic interventions targeting the Siglec–sialylated MUC1 immune axis in cancer.

## 1. Introduction

Aberrant cell surface glycosylation has emerged as a new hallmark of cancer [1,2,3,4]. The most common modifications to glycosylation include the enhanced expression of truncated or incomplete glycans, often terminated by sialic acid, and an aberrant fucosylation pattern [5,6,7]. These modifications could affect how the immune system responds to malignant cell transformations [8]. For instance, numerous studies have shown that interactions of sialylated glycans of tumor-associated MUC1 (TA MUC1) with the sialic-acid-binding lectins promote tumor immune escape, ultimately affecting the body’s antitumor immunity [9,10,11]. Concurrently, establishing connections between glycan structures and their functions as mediators of tumor progression and metastasis via immune suppression responses is of crucial importance for it to be exploited for biomedical applications. 

### 1.1. MUC1 Protein

Mucins are a family of glycosylated proteins with high molecular weight and complex molecular organization, typically characterized by a tandem repeat structure that is rich in proline (Pro), threonine (Thr), and serine (Ser) amino acids [12,13,14,15]. They are primarily categorized as gel-forming (secreted) and cell surface (transmembrane, membrane-tethered) mucins according to their structural arrangement [16,17,18]. Both mucin types function as a protective barrier for epithelial cells during homeostasis against harmful environmental factors such as toxins and pathogenic microorganisms [19]. The transmembrane mucins, which comprise MUC1, MUC3, MUC4, MUC12, MUC16, and MUC17, are anchored to the cell surface via their transmembrane domains. The short intracellular domain contains several phosphorylation sites that are shown to be involved in various signaling pathways such as phosphoinositide-3-kinase–protein kinase (PI3K/AKT), mitogen-activated protein kinase/extracellular signal-regulated kinase (MEK/ERK), epidermal growth factor receptor (EGFR), WNT/β-catenin, nuclear factor kappa B (NF-κB), and c-Jun *N*-terminal kinases (JNK/TGF-β) [20,21,22,23,24,25]. These signaling events regulate cell–cell interactions, cell growth, proliferation, differentiation, angiogenesis, and drug resistance [15,16]. Recently, signaling through the ectodomain due to the altered subcellular localization of mucins under pathological conditions has been described [26,27]. Furthermore, aberrant glycosylation of the ectodomain is known to be involved in promoting chronic inflammatory conditions that lead to malignant transformation of cells [14,28,29,30,31,32]. The important role of ectodomain in immune regulation is also well documented [33,34,35]. Thus, transmembrane mucins are well-recognized for their role in cancer initiation, progression, metastasis, and immune evasion [12,13,14,16]. The abnormal expression of transmembrane mucins is one of the primary features of adenocarcinomas, which include colon, breast, pancreatic, lung, and ovarian cancer [20,36]. More than 90% of triple-negative breast cancers (TNBC) and 80% of pancreatic ductal adenocarcinoma (PDAC), both highly aggressive cancer types, present with the aberrantly expressed MUC1 [37,38]. The high expression is often associated with poor prognosis and patient survival rate [39].

**Figure 1:**
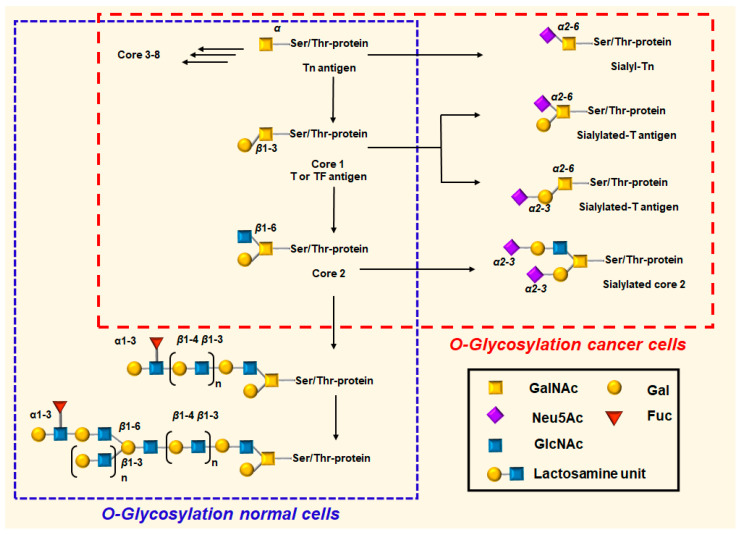
MUC1 transformation from normal to malignant phenotype in human cancers. In normal cells, MUC1 is covered with branched Galβ1-3[GlcNAcβ1-6]GalNAcα1-*O*-Ser/Thr (core 2) *O*-glycans with lactosamine extensions. The sugars form a protective and selective barrier, undertake receptor-ligand interactions, and communicate information about external cell conditions through signal transduction. The increased expression and altered density of shorter glycoforms of mucin, such as *O*-linked *N*-acetylgalactosamine (Tn: αGalNAc-), sialic acid capped Tn (sTn: αNeuNAc-2,6-αGalNAc-), Thomsen-Friedenreich (T: βGal-1,3-αGalNAc-) antigen, and sialic acid-capped T [sT: Galβ1–3(Neu5Acα2–6)-GalNAcα- and Neu5Acα2-3(Galβ1-3)-GalNAcα-] are commonly observed changes in malignant and premalignant epithelia. The expression of these TACAs is usually associated with cancer aggressiveness and poor prognosis.

MUC1 is a single-pass transmembrane glycoprotein that is expressed on the apical surface of normal epithelial cells. The structure of MUC1 consists of two subunits: The intracellular *C*-terminal domain (MUC1-C) and a large extracellular *N*-terminal tandem repeat domain (MUC1-N) [10,16]. These two domains are non-covalently linked to the smaller subunit consisting of an autocatalytic cleavage SEA-module and the transmembrane domain (TM) [40]. MUC1-N is heavily glycosylated and extends from the cell surface up to 500 nm. It contains between 25 and 125 repeats of a 20-amino acid-residue variable-number tandem repeat (VNTR) sequence HGVTSAPDTRPAPGSTAPPA, each containing five potential sites for posttranslational *O*-linked glycosylation at Ser and/or Thr residues [10]. *N*-acetyl galactosamine (GalNAc) is always the first sugar that is α-*O*-linked to the Ser or Thr residues. The addition of galactose (Gal) to GalNAc through a β1,3 linkage is catalyzed by T synthase/Core 1 synthase (core 1 β3-galactosyltransferase, β3GalT, C1GALT1). The synthesis of the disaccharide structure known as Core 1 requires the binding of T synthase to the oligomeric endoplasmic reticulum localized Cosmc protein. The addition of *N*-acetylglucosamine (GlcNAc) in β1,3-linkage to GalNAc forms a disaccharide Core 3 carbohydrate structure [4,10]. The Core 1 and Core 3 structures can both be elongated to produce the Core 2 and Core 4 trisaccharide carbohydrate structures, respectively. The primary glycan structures that have been observed in humans are core 1–4 structures. To date, eight core structures (Core 1–8 structures) have been recognized [4,10]. Long, branching glycosyl chains, can be generated by adding several poly-*N*-acetyllactosamine residues to these *O*-glycans (Figure 1) [4]. In general, the dense array of oligosaccharides adds to MUC1’s anti-adhesive properties by providing a hydrophilic surface. Termination of glycan chains by sialylation or sulfation changes results in adhesion-promoting motifs, which activate selectin and Siglec ligands [41].

During cancer progression, the long-branched glycan chains observed on normal cells become truncated, unmasking the Tn (GalNAcα-*O*-Ser/Thr, Thomsen Nouveau, CD175) and T (Galβ1,3-GalNAcα-*O*-Ser/Thr, TF, Thomsen-Friedenreich, CD176, T antigen) *O*-glycan structures. Concurrently, these changes reveal the underlying MUC1 tandem repeat polypeptide region(s) (Figure 1) [42]. Furthermore, some aberrant glycans of TA MUC1 are capped by sialic acid, either by α-2,3 linkage to Gal via ST3Gal1 or by α-2,6 linkage to GalNAc via ST6GalNAc1 sialyltransferase, respectively [43]. The most common sialylated glycan structures found on TA MUC1 are sTn (Neu5Acα2,6-GalNAcα-*O*-Ser/Thr, sialyl Tn, CD175s), 2,6-sT [Galβ1–3(Neu5Acα2–6)-GalNAcα-*O*-Ser] and 2,3-sT [Neu5Acα2-3(Galβ1-3)-GalNAc-*O*-Ser/Thr] (Figure 1). Di-sialylation of T antigen (NeuAcα2-3Galβ1-3(NeuAcα2-6)-GalNAc-Ser/Thr), in which both Gal and GalNAc are sialylated, can also occur [4]. In addition to aberrant *O*-linked glycans, the blood group sialyl-Lewis antigens, NeuAcα2,3-Galβ1,3-(Fucα1,4)-GlcNAc-R (sLeA, sialyl-Lewis A) and its isomer NeuAcα2,3-Galβ1,3-(Fucα1,3)-GlcNAc-R (sLeX, sialyl-Lewis X), glycosphingolipids, GM3 (Neu5Gc), and polysialic acid (PolySia), a homopolymer of α2,8-linked sialic acid, are generated from specific sialyl and fucosyl transferases [44,45,46,47].

### 1.2. Sialic Acid-Binding Immunoglobin-like Receptors (Siglecs)

**Figure 2:**
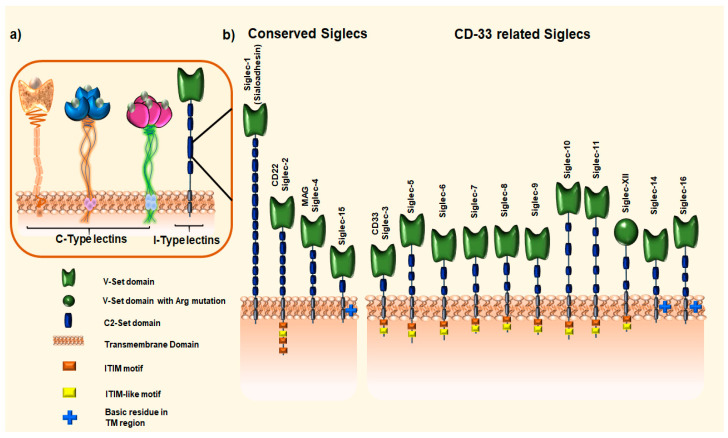
(**a**) Transmembrane lectins expressed by cells of the innate and adaptive immune systems. The C-type lectins include selectins, macrophage galactose lectin (MGL), and dendritic cell-specific intercellular adhesion molecule-3-grabbing non-integrin (DC-SIGN). (**b**) Human Siglec family. Shown are the two major Siglec subgroups: the evolutionary conserved Siglecs (Siglec-1, -2, -4, and -15), and the CD33-related Siglecs (Siglec-3, -5, -6, -7, -8, -9, -10, -11, -12, -14, and -16).

Carbohydrate-binding proteins, lectins, are the main binding partners of tumor-associated carbohydrate antigens (TACAs) of MUC1 on the cell surface. Cells of the innate and adaptive immune system express various transmembrane C- and I-type lectins (Figure 2a). The C-type lectins include selectins [48], macrophage galactose lectin (MGL) [9,49], and dendritic cell-specific intercellular adhesion molecule-3-grabbing non-integrin (DC-SIGN) [50]. Siglecs, sialic-acid-binding immunoglobulin-like lectins, are an extensive family of I-type lectins, which are immune-modulatory receptors expressed by immune cells [51,52]. In this review, we will cover the binding specificities of Siglecs to TA MUC1 and the functional role these interactions play in the modulation of the tumor immune microenvironment [53,54].

The human Siglec family consists of 15 protein members, divided based on their evolutionary conservation into two main groups: conserved (Siglec-1, -2, -4, and -15) and CD33-related Siglecs (Siglec-3, -5, -6, -7, -8, -9, -10, -11, -12, -14, and -16) (Figure 2b) [52]. Siglec-12 in humans has lost the ability to bind sialic acids due to an Arg→Cys (R122C) mutation and is, hence, designated as Siglec-XII (Figure 2b). Siglecs are type I single-pass transmembrane proteins exhibiting a modular domain architecture. The extracellular domain contains the carbohydrate recognition domain (CRD), an amino-terminal V-set immunoglobulin domain, followed by varying numbers (from 1 to up to 16) of C2-set immunoglobulin domains. The intracellular domain is engaged in signaling events [55]. Most Siglecs exhibit an inhibitory nature through the immunoreceptor tyrosine-based inhibitory motifs (ITIMs) and/or the ITIM-like motifs, which can be phosphorylated by the Src family, thereby creating a binding site for the tyrosine phosphatases SHP-1 and SHP-2 [51,56,57,58]. The immune-modulatory intracellular domain is absent from the remaining Siglecs (Siglec-1, -4, -14, -15, and -16). Siglec-1 (Sialoadhesin) and Siglec-4 (myelin-associated glycoprotein; MAG) do not engage in signaling processes. The primary mechanism of action of these Siglecs is assumed to be ligand binding [51,52]. Siglec-14, -15, and -16 contain a positively charged amino acid residue within their transmembrane domain, which enables them to attach to adaptor proteins such as DNAX-activating protein-10 (DAP10) and -12 (DAP12). These proteins contain an immunoreceptor tyrosine-based activation motif (ITAM), which can be phosphorylated in order to transmit activating signals to cells [51,55,59]. The association of Siglec-14, -15, and -16 with DAP10/12 triggers MAPK and AKT signaling pathways, leading to the activation of cells.

Structural studies by NMR spectroscopy and X-ray crystallography [60,61,62,63,64,65,66] suggest that recognition of sialoglycans is mediated by the Siglec’s *N*-terminal V-set domain, composed of two antiparallel β-sheets [67]. Within the shallow binding pocket, a key molecular interaction used by all Siglecs is a conserved essential arginine residue that forms a salt bridge with the carboxylate of sialic acid. The interaction is further stabilized by the presence of aromatic amino acids (Trp, Tyr) in the Siglecs’ binding pocket [67].

Another distinctive feature, which is highly conserved among Siglecs, is the presence of an intradomain disulfide bond between the V-set and the first C-2 domain. This and intra β-strand disulfide bonds are thought to contribute to sialic acid recognition by Siglecs. The flexible Siglecs′ loops, along with the sequence variability, are likely to provide additional underlying specificity for the glycan recognition [67]. Interestingly, the binding pockets in Siglec-1, Siglec-2 (CD22), and Siglec-8 are preformed to accommodate the ligand. On the other hand, following ligand interaction, Siglec-3 (CD33), Siglec-4, and Siglecs-7 accommodate ligands by changing the conformation of the flexible loop [55,68].

**Figure 3:**
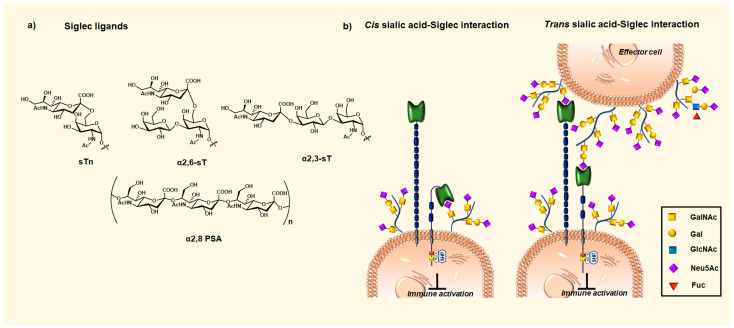
(**a**) Siglecs ligands with α2,6- (sTn, sT), α2,3- (sT), and α2,8-linkages (PSA-linear homopolymers of *N*-acetylneuraminic acid). (**b**) Cis (when present on the same cell) and trans (when present on other cells) interactions of Siglecs with sialic acid ligands lead to downstream immune inhibitory signaling.

Siglecs bind their ligands, broadly defined as sialosides with α2,6-, α2,3- and α2,8-linkages (Figure 3a), either in cis interaction, when present on the same cell membrane or trans interaction, when present on other cells (Figure 3b). Due to the high local concentration of sialic acid-bearing ligands on the immune cell surface, most Siglecs are masked by the sialic acids of their own cells in cis interactions. However, cis-ligand interactions do not necessarily prevent sialoglycan ligands present on another cell or secreted glycoproteins to trans-interact. Presumably, cis interactions are involved in setting the threshold for Siglec signaling by trans-interactions [55,69]. Siglec-1 is an exception to this rule, as it is believed that its elongated structure projects its sialic-acid-binding site away from the membrane, which reduces its cis interactions [70].

Most Siglecs have a unique specificity profile for a particular sialic acid linkage. Siglec-2 has a clear preference for α2,6-linkages; Siglec-1 prefers α2,3-linked sialic acid; and Siglec-7 and -11 prefer α2,8-linkages [71]. However, Siglecs are promiscuous receptors and bind multiple linkages. For example, Siglec-9 seems to have a preference for α2,6- and α2,3-linked sialic acid [72]. In addition, the glycan specificity is determined by the acetylation or sulfation of the sialic acids, glycan carrier backbone (lipids, proteins), and the multivalency of the interaction [71,73]. Studies with libraries of synthetic and natural glycans printed on glass arrays and novel cell-based glycan array strategies have enabled a somewhat better understanding of the Siglecs’ specificities for the natural Siglec ligands [74,75]. These studies, and particularly the cell-based glycan arrays, allowed for the evaluation of the sialic acid glycan ligands in their natural context, presented on the cell surface of particular proteins and/or lipid scaffolds [76]. The evaluation of the binding affinities of human Siglecs to a broad range of recombinant mucins and mucin-like proteins bearing different core *O*-GalNAc glycans (simple and/or complex) revealed the importance of the type and pattern of the glycan attachment for Siglecs-7 and-15 recognition [77]. However, the expected recognition of the sialyl T-MUC1 by Siglec-9 was not observed. The multivalent presentation of sialoglycans on glycoproteins, such as MUC1, or ligand clustering by glycolipids, is often needed to achieve high-avidity interactions. It has been suggested that the highly variable loops encompassing the binding site play a key role in determining the distinct fine specificities of individual Siglec family members [78]. The monovalent interactions tend to be of low affinity, typically in the millimolar range. However, these fine binding specificities are not fully elucidated, and structural information about Siglec–carbohydrate recognition is still relatively sparse. Interesting research questions remain to be answered, such as the role of multivalency either at the ligand or receptor side, their spatial organization, and the impact of the carrier on Siglecs’ diverse biological roles in the immune system.

## 2. Siglecs Immune Evasion via Sialylated MUC1 Glycans Interactions

The expression and glycosylation patterns of the MUC1 protein are altered in human carcinomas of the epithelium [10]. These unique traits of TA MUC1 lead to cell polarity loss and its localization over the entire surface of the cancer cell [13]. MUC1 is the second-most promising target for cancer immunotherapy among the top 75 tumor-associated antigens due to its widespread distribution on both primary tumors and metastases, including cancer stem cells [79]. In recent years, a wide range of naturally occurring anti-TACA antibodies, including IgM [80] and IgG [81,82], have been found in cancer patients’ serum. Their presence has been linked to improved illness prognosis [83,84] and a wealth of clinical and immunological data on MUC1 antibodies is available [85]. However, knowledge about the molecular details by which MUC1-specific antibodies recognize their targets or the role of neighboring peptide epitopes in antibody binding is limited. The lack of carbohydrate-binding specificities in most anti-MUC1 mAbs is a huge challenge for the development of MUC1-based therapeutic antibodies. Recent evidence points to the importance of tumor-associated carbohydrates in MUC1 antibody binding and for generating a robust immune response in vaccine candidates [86,87,88]. One of the best examples of such approaches is PankoMab-GEX, a humanized monoclonal antibody that is currently undergoing clinical trials for ovarian cancer. This antibody binds to a novel carbohydrate-induced conformational epitope on MUC1 (glycopeptide epitope) with substantial affinity [89]. These findings reinforce the possibility that a correct TACA presentation to the human immune system could lead to an adaptive immune response, enabling the selective eradication of TACA-displaying tumor cells.

However, the success of vaccine strategies is dependent on a better understanding of the role of the tumor microenvironment (TME) in cancer progression. The TME among, other cell types, includes diverse immune cell types. These host cells were once considered bystanders of tumorigenesis but are now known to play critical roles in the pathogenesis of cancer [90]. Whether and how tumor-associated glycans contribute to the observed immunomodulatory actions of tumors has not been extensively studied. Thus, deciphering the glycocode within the TME is the key to understanding the molecular mechanisms of these immunological events. Interactions of protein-bound glycans with their cognate lectin receptors are newly identified immune-modulatory pathways, or glyco-immune checkpoints [91].

**Figure 4:**
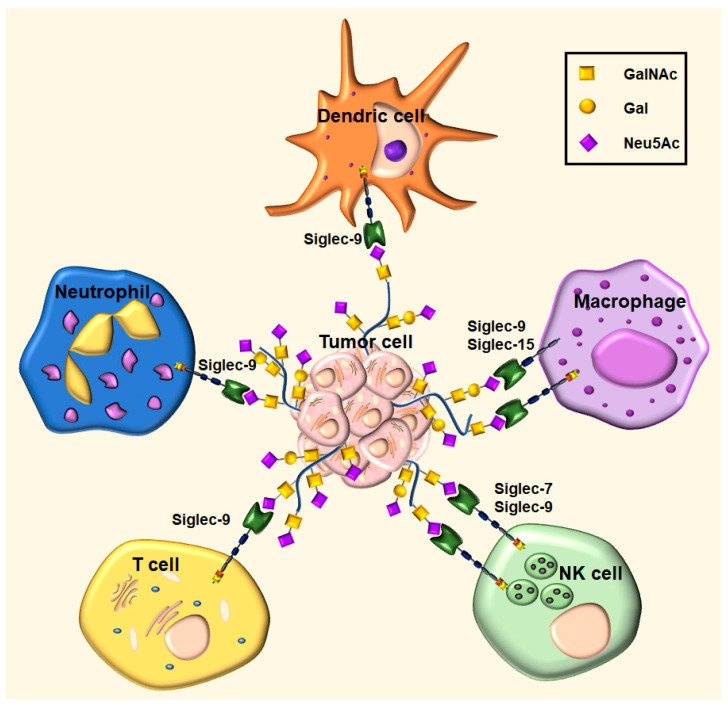
The engagement of Siglecs (-7, -9, 15) expressed on the surface of immune cells (neutrophils, macrophages, NK cells, T cells, and dendritic cells) and sialoglycans of MUC1 expressed on cancer cells results in immunosuppression in the tumor microenvironment via impaired maturation and activation of macrophages, deactivation of natural killer (NK) cells, and the formation of regulatory T cells. Thus, it is thought that these interactions are novel immune checkpoints in a similar fashion to PD-1/PD-L1 checkpoint inhibitors. The disruption of Siglec–sialylated MUC1 immune axis is a potential new target for cancer immunotherapy approaches.

TA MUC1 is one of the key carriers of the glycocode in tumor cells [10]. The tandem repeat (TR) sequence of MUC1 glycoprotein, which harbors five possible glycosylation sites, represents an ideal framework for a variety of multiple ligand arrangements for Siglecs binding (Figure 4). For instance, α2,3-sialylated MUC1 was reported to bind macrophage receptor, sialoadhesin (Siglec-1), and so may be involved in recruiting macrophages into the tumor site [92]. MUC1 carrying the monosialyl and disialyl T antigen has been identified as a counter-receptor for MAG (Siglec-4), and it was shown that their adhesive interaction might contribute to the pancreatic cancer invasion of the space surrounding a nerve [68].

The interactions of inhibitory CD-33-related Siglecs with the sialylated epitopes of TA MUC1 have been linked to the impaired maturation and activation of macrophages and dendritic cells (DCs) and are also implicated in the deactivation of natural killer (NK) cells and the formation of regulatory T cells (Figure 4) [93]. Thus, it is thought that these interactions play a central role in tumor evasion in a similar fashion to PD-1/PD-L1 checkpoint inhibitors [91,93,94].

The expression of Siglec-7 and -9 ligands on cancer cells was found to protect cancer cells from attack by NK cells, which is the first line of defense in tumor immunosurveillance. The recruitment of Siglecs-7 and Siglec-9 to the heavily sialylated tumor cells becomes advantageous for tumor survival [95,96]. The recognition of sialylated glycans on cancer cells as non- or altered-self by NK cells is blocked, and NK cell-dependent cytotoxicity is reduced or diminished (NK cell-resistant tumor cells to NK cell-mediated cytotoxicity). TA MUC1 decorated with sialylated TACAs was identified as one of the candidate ligands of Siglec-7/-9 expressed by monocytes, macrophages, and some T cells [54]. The cancer-specific MUC1 glycoforms, carrying the sialylated core 1 glycan (α2,3-MUC1-sT), could induce macrophages to display a tumor-associated phenotype (TAM), also defined as alternatively activated (M2) pro-tumorigenic macrophages, through the engagement of Siglec-9 [54]. Interestingly, MUC1-sT binding to Siglec-9 did not activate SHP-1/2 phospatases. Conversely, it was shown that there was an increased calcium flux and that the MEK-ERK pathway was active. Indeed, a recent study demonstrated the dependence of T cell activities on calcium signaling events [97]. Further research has shown that a macrophage polarized by sTn-Siglec-9 interactions is part of a specific TAM phenotype with unique functional characteristics, which is frequently linked to a low prognosis for patients with breast cancer. These MUC1-sTn-induced macrophages can activate immune-suppressive neutrophils, which can impede antitumor T-cell responses and promote invasion and metastasis [54]. Since Siglec-9 is widely expressed in human neutrophils, anti-Siglec-9 antibodies that block the trans interaction have been shown to enhance neutrophil activity against tumor cells in vitro [98]. Furthermore, enhanced antibody-dependent tumor cell cytotoxicity by neutrophils was achieved by targeting the Siglec–sialylated MUC1 immune axis, either by blocking Siglec-9 or by reducing tumor cell sialylation [99]. It has recently been shown that sialoglycans on pancreatic ductal adenocarcinoma cells (PDAC) engage Siglec-7 and Siglec-9, which can cause monocytes to differentiate into macrophages with an immune-suppressive phenotype [100]. Increased expression of α2,3 sT antigen and monocyte-derived macrophages are the main prognostic markers of low survival in PDAC patients. According to a recent study, TAMs in PDAC express Siglec-15, an immune checkpoint molecule with sequence homology to programmed cell death ligand 1 (PD-L1). The binding of Siglec-15 to tumor-associated α2,6 sTn antigen leads to the suppression of the immune system. Despite having an ITAM in its intracellular domain, Siglec-15 has been demonstrated to be a strong inhibitor of CD8+ T lymphocytes. As a result, Siglec-mediated immunosuppression is an essential modulator of tumor growth [101].

Dendritic cells are other crucial antitumor immune response mediators and play a key role in the effectiveness of immunotherapies [102]. Recent studies have revealed an important role of Siglecs receptors in T cell biology, such as modulation of DC activation and antigen presentation, generation of antigen-specific regulatory T (Treg) cells, and prevention of the formation of effector CD4+ and CD8+ T cells [103,104,105]. Human monocyte-derived dendritic cells expressing sialoglycans suppressed immune cell activation via Siglec-7 and Siglec-9 [106]. Siglec-9 mediates inhibition of DCs ability to differentiate and activate [107] through interaction with sialylated MUC1 [108]. Furthermore, sialoglycans have been demonstrated to promote high-avidity interactions between DCs and CD8+ T cells [109].

## 3. Targeting MUC1–Siglec Axis in Cancer

Perturbations of the Siglec–sialic acid immune axis lead to an immunoregulatory pathway imbalance, followed by the development of a wide range of diseases, including cancer. Hence, strategies to modulate the sialic acid–Siglec axis may have significant therapeutic potential for cancer treatments [67]. In addition, it is becoming increasingly evident, from several basic and translational studies, that interference with sialoglycan–Siglec interactions could represent a new immune checkpoint, similar to PD1/PD-L, and a potential new target for cancer immunotherapy [91,93,94]. By disrupting the Siglec–sialoglycan interactions, the response of immune cells can be modulated to enhance anti-cancer immunity. This can be achieved by using sialic acid mimetics, antibodies, or glycan-modifying drugs (which change the synthesis and expression of the sialic acid glycocalyx). Nevertheless, each approach has its own difficulties. A better understanding of the structural diversity of Siglecs and their specificities for sialoglycan ligands is necessary before they can be targeted to increase antitumor immunity.

The Siglec–sialylated MUC1 immune axis is a potential new target for cancer immunotherapy approaches.

### 3.1. Sialic Acid Mimetics (SAMs) as High-Affinity Ligands for Siglecs

**Figure 5:**
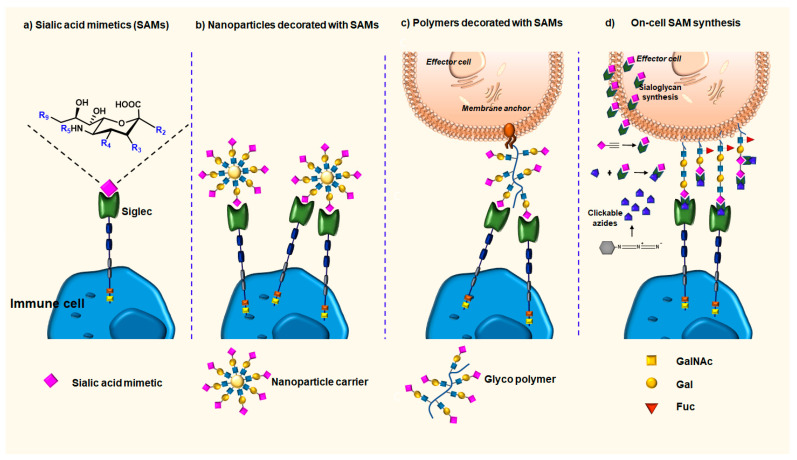
(**a**) Carbon groups (C-) in the sialic acid backbone that could be substituted to design high-affinity SAMs are highlighted in blue; (**b**) nanoparticle carriers decorated with sialic acid mimetics for high-affinity binding to Siglecs; (**c**) glycopolymers that target membranes and can bind to Siglec on immune cells; (**d**) creation of a highly reactive cell glycocalyx (clickable sialic acid-azide approach) for Siglecs.

The sialic acid mimetics (SAMs) are small-molecule inhibitors that have the ability to target specific Siglec ligand binding sites (Figure 5a). Their discovery stems from the early findings that sialic acid structural characteristics are crucial for Siglec binding. The first SAMs developed focused on Siglec-2 as a target [110,111]. These studies facilitated further research into establishing the contribution of substituents at different carbons (C2-C5 and C9 positions) of the sialic acid backbone to binding to Siglec-2 and also other members of the Siglecs family [112]. Compared to the weak monovalent interaction between Siglecs and natural sialosides (0.1–3 mM), SAMs exhibit higher affinity (low micromolar range) and a somewhat improved specificity range [113]. However, additional improvements in affinity and, particularly, specificity of SAMs are desired to better comprehend the biological role and potential therapeutic utility of the individual Siglec family members. The combinatorial chemistry approaches combined with the computational optimization of the hit compounds may facilitate the identification of high-affinity ligands. The fine binding specificities may be dictated not only by the glycan but also by the glycan carrier.

Furthermore, to outcompete natural ligands, high-avidity binding is required for clustering of the receptor to trigger Siglec signaling. A promising alternative is the use of a multivalent display of sialic acid mimetics, either by linking SAMs to nanoparticles or polymers, or by modifying the cell glycocalyx of the living cells via biorthogonal synthesis [113,114,115]. Liposomes, gold nanoparticles, dendrimer/poly lactic-co-glycolic acid (PLGA)-based nanoparticles, and other SAM-decorated nanoparticles (Figure 5b) have been used to either transport cargo to Siglec-expressing cells [116] or to stimulate Siglec signaling through the use of glycan-decorated nanoparticles [117,118]. Lately, phospholipid-tailed polymers containing naturally occurring sialosides (α2,3- or α2,6- SiaLacNAc, GD3, or sialyl LewisX) have been synthesized to facilitate their integration into the cell membrane [95,119]. Cells bearing these glycopolymers, mucin mimics, (Figure 5c) exhibit a significant increase in binding to Siglec-7 and were protected from being killed by Siglec-7+ NK cells. Subsequent investigation revealed that the phosphorylation of Siglec-7 and the consequent recruitment of SHP-1 occurred at the same time as the suppression of the cytotoxic activity of NK cells. Together, these findings suggest that soluble or membrane-incorporated polymers may function as carriers of SAMs, enabling them to reach Siglecs on immune cells and trigger their immunosuppressive signaling. Further studies are needed to demonstrate their biological potential in in vivo cancer models.

Glycoengineering utilizes bioorthogonal chemistry approaches to modify sialic acids on the cell surface to enhance interactions and increase binding affinity for Siglecs (Figure 5d) [120]. The high-affinity ligand for Siglec-7 was identified from a screening of a large sialoside library prepared by the click chemistry approach [121]. The ligand-bearing liposomes, warranting the multivalent ligand presentation, were then assessed for binding to Siglecs-expressing cells. Only Siglec-7 expressing cells were positive for binding, thus indicating the potential therapeutic application. The advancement of bioorthogonal chemistry approaches is further paving the way for modulation of the sialic acid–Siglec axis in vivo through metabolic engineering [122]. This platform may enable identification of the fine binding specificity of Siglecs in their natural context and guide the development of novel, more potent, and specific glycan-based therapeutics.

### 3.2. Anti-Siglecs Antibody-Based Approaches

Targeting Siglec receptors with antibodies and antibody-based therapies has been explored for several years as a potential anti-cancer therapeutic approach [114,123]. Recent findings of the sialoglycan–Siglec interactions, representing a new immune checkpoint, further facilitated the use of anti-Siglec antibodies to modulate the response of immune cells rather than depleting them. These antibody-based approaches include unconjugated antibody and/or antibody–drug(toxin) conjugates (ADCs), bispecific antibodies (BsAbs), and chimeric antigen receptors (CARs) [124]. Anti-Siglec-targeted antibodies have not demonstrated sufficient activity in the cancer setting [125]. The ADCs targeting Siglec-2 (CD22) [126] and Siglec-3 (CD33) [127] have shown more promise; however, they carry cytotoxicity problems [128,129]. BsAbs have been proven clinically effective and approved for the treatment of hematologic malignancies but have shown limited clinical application in solid tumors due to the suppressive tumor microenvironment. A new bispecific antibody (BsAb) that engages T cells, and targets both MUC1 and CD3, has been demonstrated to effectively increase T cell cytotoxicity, cytokine release, and activation [130]. Novel therapeutic strategies, such as CAR T cells, that aim to interfere with the Siglec-sialic acid immunological axis are starting to show early promise in clinical studies [131].

#### 3.2.1. Anti-Siglecs Antibodies

**Figure 6:**
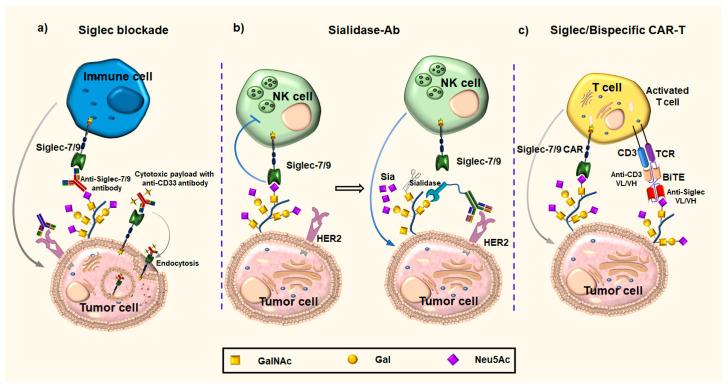
Antibody-based approaches. (**a**) Anti-Siglecs antibodies block the Siglec–ligand interaction and activate the immune cell attack on the cancer cell; anti-Siglecs antibody–drug(toxin) conjugates are comprised of two parts; the anti-Siglecs antibody portion of the drug targets Siglecs on the surface of cancer cells, leading to the internalization of the antibody and the drug and successive release of the cytotoxic payload within the cancer cell. (**b**) Anti-Siglecs antibody against HER2–sialidase conjugates can remove sialic acid from surface of glycans and restore the immune cell response. (**c**) Chimeric antigen receptors (CARs); (left) Siglec-7/-9 CARs can recognize and eradicate cancer cells through binding to sialylated glycans. (Right) Activated T cells are linked to cancer cells via anti-Siglec BiTEs, which cause the cancer cell to be eradicated.

Considering a variety of human epithelial cancers overexpressing Siglec-7 and -9 receptors on their cell surface, anti-Siglec antibody approaches are explored to boost an antitumor immune response (Figure 6a) [132,133]. The success of targeting these Siglecs depends on the availability of highly specific antibodies for Siglec-7 and -9, respectively. For example, Siglec-7 and 9 share 80% sequence similarity but still differ in their ligand-binding domain. In addition, Siglec–sialic acid interactions are important immune-negative checkpoints against autoimmunity [134]. It has been shown that the engagement of Siglec-7 and -9 receptors attenuates both myeloid and lymphoid antitumor responses [53,95,96,98,108,135]. Several studies showed that Siglec-9 is an attractive target for boosting a T cell antitumor response [135,136]. Similarly, blocking antibodies against Siglec-9 improved neutrophil-mediated cytotoxicity by therapeutic antibodies [99]. Siglec-7 and Siglec-9 have also been defined as potential targets to improve NK cell antitumor activity [95,96]. The development of a humanized immunocompetent murine model allowed testing of the therapeutic potential of anti-Sigec-7- and anti-Siglec-9-targeting antibody clones for their ability to block the interaction between Siglecs-7 and -9 and their ligands on tumor cells [132]. Furthermore, the use of engineered anti-Siglec-7 and -9 blocking antibodies significantly reduced tumor burden in this mouse model [132]. These findings suggest that anti-Siglec-7 and Siglec-9 antibodies can inhibit the antitumor immune response, possibly by preventing the polarization of macrophages into TAMs, in agreement with the in vitro data by Beatson et al. [53]. The humanized antibodies against Siglec-7 showed the ability to enhance NK activity against ovarian cancer cell lines. Furthermore, ovarian cancer-challenged mice showed improved survival rates when treated with an anti-Siglec-7 monoclonal antibody (DB7.2) [137]. The humanized and high specific monoclonal antibody against Siglec-9 has been developed by immunizing mice with Siglec-9-encoding DNA and Siglec-9 protein [138]. The in vitro and in vivo assays showed that the lead antibody (8A1E9) enhances anti-tumor immune activity. In mouse models that constitutively express Siglec-15, it has been demonstrated that the anti-Siglec-15 (NC318) humanized IgG1 monoclonal antibody inhibits tumor growth and prevents metastasis by blocking Siglec-15 interactions with the tumor microenvironment [101]. Promising results in solid tumors, treated with NC318 alone or in combination with pembrolizumab, an anti-PD-1 antibody, were reported [139].

Immunomodulators of sialoglycan–Siglec interactions are promising candidates for improving anti-cancer immunity. Several anti-Siglecs antibodies that either inhibit the anti-tumor immune response or augment the immune response against tumors are currently in preclinical or clinical trials [140]. However, further studies are warranted considering the Siglecs’ diverse biological roles in the immune system.

#### 3.2.2. Anti-Siglecs Antibody–Sialidase Conjugates

A novel strategy for interrupting the Siglec–sialic acid immune axis includes selective desialylation of the glycan ligands on cancer cells. This can be achieved by combining sialidase, an enzyme that efficiently removes sialic acid from glycans, with antibodies that target sialylated epitopes on tumors (Figure 6b). The technology termed EAGLE (enzyme-antibody glycan-ligand editing) was originally developed by coupling the human epidermal growth factor receptor 2 (HER2)-specific antibody to a sialidase from Vibrio cholerae [141] and Salmonella typhimurium [140,142] that selectively removed diverse sialoglycans from breast cancer cells, leading to enhanced immune cell infiltration and activation, as well as prolonged survival, in mouse models. It has been suggested that this approach specifically cuts off the sialic acid ligands that are bound by Siglec-7 and Siglec-9 [141]. This technology led to the development of the first glyco-immune checkpoint inhibitor, E-602, currently evaluated in clinical trials alone and in combination with cemiplimab (anti-PD-1 antibody) in patients with advanced cancers (NCT05259696). It remains to be seen if this approach can be applied to other than HER-2-positive cancers. Overexpression of HER-2 occurs only in 15–30% of breast cancers and 10–30% of gastric/gastroesophageal cancers. Identification of cancer-specific antigens for many other cancers is still lacking, hindering the widespread use of cancer immunotherapies. Furthermore, additional studies are needed to determine if partial or complete tumor desialylation is required for diverse immune cell types to infiltrate the tumor microenvironment and whether interactions of exposed galactose residues with their cognate receptors, galectins, should be taken into account. Therefore, more research should be conducted, considering galectins’ various functions in the development of cancer and the immune system’s response to cancer [143,144].

#### 3.2.3. Chimeric Antigen Receptors

CAR-T cells are T cells that have been genetically engineered to express T cell receptor (TCR) signaling components in their cytoplasmic/transmembrane regions and chimeric antigen receptors (CARs) in their extracellular regions (Figure 6c). Bispecific T cell engagers (BiTEs), based on two fused single-chain mAbs, is an emerging therapy for the management of hematologic malignancies (Figure 6c). It has been demonstrated that CAR-T cells expressing anti-CD33 and anti-CD3, and anti-CD22 and anti-CD33 CARs, have strong antitumor effects in patients with leukemia and lymphoma [145,146,147]. Bispecific CAR-T cells that target both CD22 and CD19 have been shown in recent studies to be a promising therapeutic approach for the treatment of hematologic malignancies [148]. However, this immunotherapy has demonstrated limited efficacy in solid tumors.

CAR T-cell therapy, employing T-cells that were reprogrammed to express the Ig domains from Siglecs V- and C2-set, is being applied to target the Siglec-sialic acid immune axis. Enhanced cytotoxicity against hypersialylated tumors was shown by Siglec-7 and Siglec-9 CAR-T cells in xenograft mouse models [149]. Nonetheless, whether Siglec-CAR-engineered T cells are useful as a therapeutic intervention for solid tumors is still unknown. Recent studies have demonstrated the effectiveness of CAR-macrophages and CAR-NK cells in the removal of tumors [150,151,152]. Although a promising new approach, CAR immunotherapy needs more research due to its unfavorable side effects, which include CNS toxicity, cytokine release syndrome, and off-target effects.

Targeting MUC1 glycans is the other cutting-edge strategy. Numerous researchers have produced and altered CAR T cells that specifically target MUC1, and they have examined the effectiveness of these cells in various cancer models. CAR T cells that target hypoglycosylated MUC1 have demonstrated remarkable efficacy against triple-negative breast cancer model (TNBC) [153,154] and head and neck cancer [154]. Additionally, CAR T cells targeting hypoglycosylated MUC1 in hematological and pancreatic cancers showed encouraging results [155]. The anti-Tn-MUC1 CAR T cells exhibited target-specific cytotoxicity and effective tumor growth inhibition. The therapeutic potential of CAR T cells targeting Tn-MUC1 in cancer therapy is highlighted by these findings.

### 3.3. Glycan Modifying Agents for Altering Synthesis and Expression of the Sialic Acid Glycocalyx

**Figure 7:**
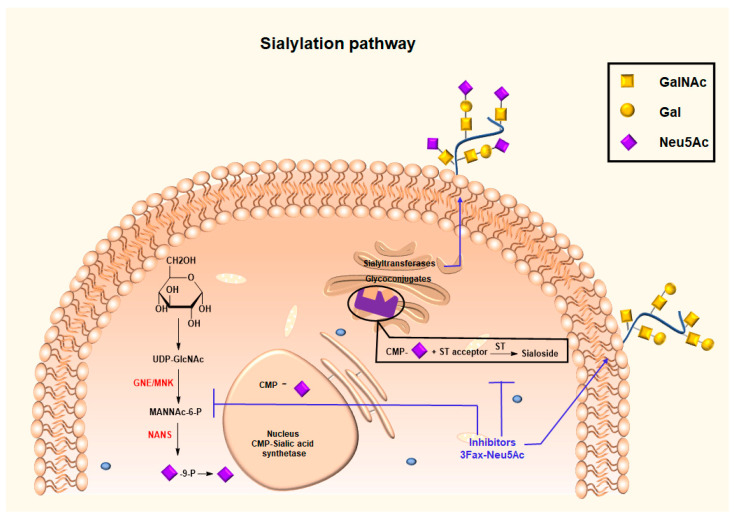
The use of small-molecule metabolic inhibitors, such as the fluorinated sialic acid analogue 3Fax-Neu5Ac, to obstruct the synthesis of sialylation pathway. As a result, the cell surface-expressed glycans are not capped with sialic acid, and their interaction with Siglecs is blocked.

Another strategy to disrupt the Siglecs–sialoglycan immune checkpoint is to therapeutically reduce the density of sialoglycan on tumor cells and in the tumor microenvironment. This can be accomplished by using inhibitors of the sialic acid synthesis pathways. These include inhibition of the cytidine monophosphate (CMP)-sialic acid transporter and inhibition of sialyltransferase enzymes (Figure 7).

In response, synthetically produced and chemically altered sialic acid analogues were developed. Due to the inability of these glycomimetics (sialyltransferase inhibitors) to bind to the glycan chain, they accumulate in the Golgi apparatus, causing a negative feedback loop for de novo sialic acid synthesis and ultimately mediating a reduced density of sialoglycans in the tumor glycocalyx [156,157]. As established by Rillahan et al. [156] the 3-fluoro sialic acid mimic prodrug (P-3Fax-Neu5Ac) can be converted into the active inhibitor CMP-3Fax-Neu5Ac, which then shuts down the de novo synthesis of sialic acids by feedback inhibition. The use of this global metabolic inhibitor of sialylation exhibited an immune-mediated delay of tumor growth in in vivo tumor models for melanoma and neuroblastoma research and prevented metastasis formation in a murine lung metastasis model [158,159]. However, these inhibitors have been shown to be harmful for liver and kidney function when applied in vivo [160]. The targeted delivery of P-3Fax-Neu5Ac using nanoparticles was explored to overcome these side effects [159]. The renal toxicity was still noted at higher doses, thus highlighting the need for better-tolerated versions of these types of inhibitors for use in vivo. Recently, more potent C-5 carbamate-modified 3-fluoro sialic acid mimetics were identified [157]. These novel inhibitors are more efficiently converted to their CMP counterparts, consequently reaching higher effective concentrations capable of inducing persistent inhibition of α2,3 and α2,6-linked sialylation, respectively [157]. However, these studies were performed in human and mouse cancer cell lines.

Furthermore, several naturally occurring compounds possess the ability to hinder particular sialyltransferase activity. These include ginsenosides, which can inhibit both α-2,3- and α-2,6-sialylation [136], flavonoids, which can inhibit ST6GAL1 [161], lithocholic acid, which acts against ST3GAL1, and soyasaponin I, which obstructs ST3GAL1 [162,163]. The soyasaponin I analog, Lith-O-Asp, exhibiting broader specificities for sialyltransferases (ST3GAL1, ST6GAL1, and ST3GAL3) was shown to suppress metastasis in an in vivo model [164,165]. Moreover, a highly potent cyclopentane-based inhibitor of ST6GAL1 was reported [166]. The limitations and challenges of designing selective, potent, and cell-permeable sialyltransferase inhibitors have hindered their further development into clinical trials.

## 4. Conclusions

Although early clinical applications of Siglec-targeted therapy are promising, there are lessons to be learned from the limited efficacy of PD-1/PD-L1 inhibitors. Challenges such as the diversity of Siglecs function and the complexity of sialome have to be overcome, and new interdisciplinary research efforts are needed to facilitate structural and biological studies. Siglecs play several key roles in normal physiology, including immunomodulation and dampening of the innate and adaptive immune responses against “self” molecular sialic acid-bearing ligands. In cancers, tumor cells exploit the normal functions of Siglecs to down-regulate an immune response against them. In order to impede tumor immune evasion, it is of crucial importance to understand the fine-intricate nature of immune cells to either augment the anti-tumorigenic or minimize the pro-tumorigenic effects. The Siglec–sialylated MUC1 immune axis is targeted by using sialic acid mimetics, antibodies, or obstructing the synthesis of the sialylation pathway in cells. Some of these approaches demonstrated promising preclinical data and moved forward to clinical trials. Nevertheless, potential toxicities and efficacy will need to be carefully evaluated. To fully achieve the clinical applicability of targeting the Siglec–sialylated MUC1 immune axis, the fine tuning of activating and inhibitory signaling and the integration of precision medicine will be essential. The success and broader application of personalized immunotherapies have been limited due to the heterogeneity in production, secretion, and glycosylation patterns of MUC1 and the distribution of Siglecs receptors among various tumor types and between patients. The multidisciplinary approach, combining the application of novel genomics and proteomic technologies with artificial intelligence (AI), warrants advancing cancer immunotherapies against different cancers and further improvements in tailoring patient-specific therapies.

## Data Availability

Data analyzed in this study were a re-analysis of existing data, which are openly available at locations cited in the reference section. Any further questions can be addressed to the corresponding author.
